# Evaluation of *Aloe elegans* Mucilage as a Suspending Agent in Paracetamol Suspension

**DOI:** 10.1155/2021/5058372

**Published:** 2021-07-31

**Authors:** Gebremariam Woldu, Berhe Baymot, Desta Tesfay, Gebre Teklemariam Demoz

**Affiliations:** ^1^Department of Pharmacy, College of Health Sciences, Aksum University, Aksum, Ethiopia; ^2^Department of Biomedical Science, College of Health Sciences, Aksum University, Aksum, Ethiopia; ^3^Department of Pharmacy, Mekelle University, Mekelle, Ethiopia

## Abstract

**Background:**

There are various natural excipients which have been used as suspending agents in pharmaceutical suspensions due to the presence of mucilage in their specialized cells and their capacity to form a colloidal gel in an aqueous medium.

**Objective:**

The purpose of this study was to evaluate the suspending capacity of *Aloe elegans* mucilage in suspension formulations.

**Materials and Methods:**

*Aloe elegans* mucilage (AEM) was evaluated as a suspending agent in comparison with xanthan gum (XG) in paracetamol suspensions at 1, 2, 3, 4, and 5% (*w*/*v*) concentrations. The resulting suspensions were evaluated for their sedimentation volume, apparent viscosity, flow rate, rate of redispersibility, pH, assay, and dissolution profile.

**Results:**

The volume of sedimentation, apparent viscosity, and redispersibility rate of the formulations were significantly increased (*p* < 0.05), with the concentration of the suspending agents. Meanwhile, the apparent viscosity for all formulations has significantly decreased (*p* < 0.05) with an increase in shear rates. Volume of sedimentation, apparent viscosity, and redispersibility degree of the formulations prepared with AEM were significantly (*p* < 0.05) lower than XG-containing formulations at the same concentration. Nevertheless, the sedimentation volume of all formulations with AEM was significantly (*p* < 0.05) higher than the suspension without any suspending agent. With regard to drug content and pH values, all formulations showed an acceptable result with the standards. All formulations showed a release of greater than 85% of drug content within 45 min.

**Conclusion:**

*Aloe elegans* mucilage could have a potential to be utilized as an alternative suspending agent in pharmaceutical suspensions.

## 1. Introduction

Pharmaceutical suspensions are thermodynamically unstable liquid dosage forms, which consist of insoluble solid particles dispersed in a liquid continuous phase [1]. The solid particles in suspensions will eventually settle down, because of their sizes (usually greater than 1 *μ*m) which are sufficiently large enough for sedimentation [2]. Formulation of stable suspensions requires the use of excipients that help to hold particles of the active ingredient suspended in a uniformly distributed manner for a noticeably real-looking length of time [3]. Therefore, it is essential to include suspending agents in suspension formulations to reduce the rate of settling and to allow uniform dosing of the drug to the patient [4].

Suspending agents are substances that are used to keep the stability of the suspension through the rising viscosity of a suspending medium, reduced sedimentation rate, increased sedimentation volume, convenient redispersibility, and diminished compact mass formation [5, 6]. The sources of suspending agents can be natural (mucilage), synthetic (polyvinyl pyrrolidone), or semisynthetic (cellulose derivatives) polymers [7]. However; the use of natural polymers like mucilages for pharmaceutical applications as a suspending agent is fascinating because they are cheap, effortlessly available, nontoxic, amenable to chemical modifications, and potentially biocompatible [8]. Moreover, natural polymers have a wide range of applications in different fields like locust bean gum-based hydrogels that are reported to be utilized as an alternative and promising adsorbent for the treatment of effluents containing water-soluble cationic dye-brilliant green [9], sodium alginate stabilized silver nanoparticle-silica nanohybrid that has various antibacterial applications in biotechnology and biomedical fields [10], and *κ*-carrageenan-based silver nanoparticle nanocatalysts that exhibit high catalytic degradation and mineralization of industrially important organic dyes such as rhodamine B and methylene blue, with a degradation efficiency of 100% in a very short period of time [11].

In recent times, gums and mucilages extracted from plant sources have evoked incredible interest due to their diverse applications in pharmaceutical formulations of both solid and liquid dosage forms serving as thickeners, suspending agents, emulsion stabilizers, binders, and film formers [12].

The genus *Aloe* which belongs to the Liliaceae family consists of around 600 species, and their main distribution is found to be in Africa, the Arabian Peninsula, and the islands of the western Indian Ocean [13]. Right now, there are 46 species of *Aloe* found in Ethiopia and Eritrea [14]. Among these species, *Aloe elegans* is one of the indigenous plants in Ethiopia and found in most regions of the country like the Tigray, Welo, Gojam, Shewa, and Harerge regions [14]. Generally, the leaves of the *Aloe* species have two major parts, the outer green rind that consists of the latex and the inner colorless parenchyma that comprises the mucilage [15]. The mucilage is a clear jelly substance that contains polysaccharides and water [16]. A study done in Nigeria showed that the mucilage obtained from *Aloe vera* had good suspending capacities as compared to acacia and sodium carboxy methyl cellulose in zinc oxide suspensions [17].

Due to the fact that Ethiopia is well known for numerous *Aloe* species, including *Aloe elegans* that produces mucilage, the suitability of *Aloe elegans* mucilage as a suspending agent has not been studied so far. Thus, the present study is aimed at evaluating the mucilage from *Aloe elegans* as an alternative or complementary natural suspending agent, to be utilized in pharmaceutical suspension preparations.

## 2. Materials and Methods

### 2.1. Materials

The materials used include xanthan gum and hydrochloric acid (HCl), both of which are from unival, Germany. Methyl paraben, propyl paraben, and NaCl are from Zhejiang, China. Polysorbate 80, propylene glycol, and sodium hydroxide are products of B.M.P. Bulk Medicines & Pharmaceuticals GmbH, Germany. Glycerin (Dubai and Hubei Hungyanphar) was obtained from Addis Pharmaceutical Factory (APF). All the materials utilized in this experiment were pure and found to be of analytical grade.

### 2.2. Methods

#### 2.2.1. Extraction

We collected the fresh leaves of *Aloe elegans*, and we removed its spines located along the leaf margins using a sharp knife. Then, we carefully separated the gel from the leaf by introducing the knife into the mucilage layer. We homogenized the gel (1 : 1) with distilled water using an electrical blender (SEVEN STAR, Germany). The homogenized gel was then macerated for 24 hours (hrs), vibrated on an orbital shaker (Bibby Sterilin, UK), and boiled at 70°C for 30 min (minutes) in a hot plate (Bibby Sterilin, UK), and it was then put aside for 2 hrs in order to release the mucilage. The extract was filtered through 8 folds of muslin cloth to remove the mark from the filtrate. The mucilage was then isolated from the aqueous filtrate by the addition of 96% ethanol at a ratio of 1 : 3 [18]. Finally, the mucilage was dried in an oven (Genlab, Widnes, England) at 50°C, powdered, and passed through a 224 *μ*m sieve; indeed, we stored the powder in a desiccator for further use.

#### 2.2.2. Physicochemical Investigation of the Mucilage

*(1) Micromeritic Properties*. The mucilage has the following micromeritic properties:

1. Bulk density and tapped density

In determining the bulk density, 50 g powder was placed in a 250 ml clean, dry measuring cylinder, and the volume occupied by the sample without tapping was noted. In order to determine the tapped density of the mucilage, the powder was tapped using a tap density analyzer 50 times for 3 min [19].

(1) $$\begin{array}{c} \text{Bulk density}=\frac{\text{weigt of the sample powder }\left(\text{g}\right)}{\text{volume of the saple powder }\left(\text{ml}\right)},\;\\ \text{Tapped density}=\frac{\text{weigt of the sample powder }\left(\text{g}\right)}{\text{volume of the saple powder after tapping }\left(\text{ml}\right)}. \end{array}$$

(2) Carr's compressibility index and Hausner's ratio

The mucilage's Carr's compressibility index and Hausner's ratio were determined based on the methods used elsewhere [20]:

(2) $$\begin{array}{c} \text{Car}{\text{r}}^{\text{'}}\text{s index }\left(\%\right)=\frac{\text{tapped density}-\text{bulk density }}{\text{tapped density}}\times 100,\\ \text{Hausne}{\text{r}}^{\text{'}}\text{s ratio}=\frac{\text{tapped density }}{\text{bulk density }}. \end{array}$$

(3) Angle of repose

We determined the angle of repose of the mucilage powder using the funnel method [21]. The funnel was fixed using a retort stand with its bottom orifice of 10 cm above the table surface. Then, we filled the funnel with mucilage powder and let it pour on a graph of paper placed on the table. Lastly, we determined the average radius (*r*) and height (*h*) of the powder piles formed on the table, and the angle of repose was calculated as follows:

(3) $$\begin{array}{c} \text{Angle of repose }\left(\theta \right)=\tan^{-1}\left(\frac{h}{r}\right). \end{array}$$

*(2) Swelling Power*. To determine the swelling power of the mucilage, we dispersed 1 g of the mucilage in 100 ml of water. Next, we placed the dispersion in a thermostatically controlled water bath at a temperature of 20, 30, 40, 50, and 60°C. Then, after we have shaken the dispersion for 10 min, it was left to cool at room temperature and centrifuged at 3000 rpm for 15 min. Finally, we collected the supernatant using a preweighed evaporating dish and dried it in an oven at 105°C for 3 hrs until constant weight was maintained [22].

(4) $$\begin{array}{c} \text{Swelling power}=\frac{\text{mass of the precipitated mucilage}}{\text{mass of the mucilage taken}-\text{mass of dried supernatant}}. \end{array}$$

*(3) Apparent viscosity*. The apparent viscosity of the mucilage was determined by a viscosity tester using spindle number 2 at shear rates of 20, 30, 50, 60, 100, and 200 rpm [23].

*(4) pH*. We prepared different dispersions (1, 3, 6, and 9% *w*/*v*) by mixing the mucilage powder with distilled water and measured the pH of the dispersions using a calibrated digital pH meter at room temperature [24].

*(5) Phytochemical screening*. The mucilage was counterchecked for the presence of secondary metabolites such as anthraquinones, carbohydrates, steroids, alkaloids, saponins, phenols, tannins, flavonoids, and coumarins [25].

#### 2.2.3. Preparation of Suspension

Paracetamol suspension formulations were prepared using xanthan gum (XG) and AEM as suspending agents. The formulations were grouped into two as FX- (for XG) and FA- (for AEM) containing formulations, and each group has five formulations (FX1-FX5 and FA1-FA5). The digits 1, 2, 3, 4, and 5 represent the concentrations of the suspending agents. Primarily the suspending agents and Tween 80 at a concentration of 0.4% (*w*/*v*) were dispersed in distilled water which contains methyl paraben (0.18% *w*/*v*) and propyl paraben (0.02% *w*/*v*). Then, 3.2% (*w*/*v*) paracetamol was wetted with propylene glycol 5% (*w*/*v*), and glycerin 5% (*w*/*v*) was added to the vehicle and stirred continuously till uniform dispersion was obtained [26].

#### 2.2.4. Evaluation of Suspensions

*(1) Sedimentation Volume of the Suspensions*. Fifty ml of each formulation was transferred to a 100 ml graduated cylinder and allowed to stand for a month at room temperature without agitation. The volume occupied by the solute was then recorded every day for seven days and then every week for 3 consecutive weeks [27].

1. Effect of electrolyte concentration on sedimentation volume

To 50 ml of suspension containing 3% of the suspending agents, different concentrations of NaCl (10^−4^, 10^−3^, 10^−2^, and 5 × 10^−2^ molar) were added separately during preparation and the sedimentation volumes of the respective formulations were noted every day for seven consecutive days [28].

(2) Effect of pH on sedimentation volume

The effect of pH (at pH values of 2, 6.5, 8, and 10) on the suspension was assessed by treating 50 ml of the suspension containing 3% of AEM and XG as suspending agent with 0.1 N HCl and 0.1 N NaOH. Each of the preparation was poured into a graduated measuring cylinder, and the sedimentation volumes were noted every day for seven successive days [28].

*(2) Apparent Viscosity of the Suspensions*. The apparent viscosities of the formulations containing AEM and XG as suspending agents were determined at room temperature at different shear rates (20, 30, 50, 60, 100, and 200 rpm) using a viscosity tester (R2) [29].

*(3) Flow Rate of the Suspensions*. The flow rates of the suspensions were measured as the time required for 10 ml of the suspension to flow through a pipette [27].

*(4) Redispersibility Rate of Suspensions*. A fixed volume of each suspension (50 ml) was kept in a measuring cylinder which was stored at room temperature for various time intervals (one week and four weeks). The measuring cylinders were moved upside down manually, and the number of times the cylinders were inverted until the sediment was uniformly redispersed was noted [30].

*(5) pH of the Suspensions*. The pH value usually represents the acidity or alkalinity of an aqueous solution. The pH of a particular suspension was determined by using a pH meter [31].

*(6) Particle Size*. The particle sizes of the formulated suspensions were measured using a microscope. Drops of suspensions were separately put on slides and placed on the stage of the microscope [31].

*(7) Assay of the Suspensions*. The active drug within the formulations was determined by a UV visible spectrophotometer (Shimadzu, Japan) [29].

*(8) Dissolution Profile of the Suspensions*. *In vitro* dissolution profiles of the suspensions were studied according to the 2013 specifications of BP using a dissolution tester (paddle method) (Pharma Test, Germany). The temperature of the dissolution medium (495 ml of 0.1 N HCl) was maintained at 37 ± 1%^o^C and at 25 rpm. From each formulation, a 5 ml suspension was injected into the dissolution medium with the aid of a 10 ml syringe. Then, 5 ml aliquots were withdrawn at prescheduled time intervals (5, 10, 15, 20, 30, and 45 minutes) from the dissolution medium. The amount released (%) from the suspensions was measured using a UV spectrophotometer at *λ* max of 242 nm by using 0.1 N HCl as a blank [32].

#### 2.2.5. Statistical Analysis

Statistical analysis was carried out using analysis of variance (ANOVA) with Origin 8 (OriginLab Corporation, USA) software. At 95% confidence interval, *p* values less than or equal to 0.05 were considered statistically significant. All the data measured and reported in this study were averages of a minimum of triplicate measurements, and the values are expressed as mean ± standard deviation (SD).

## 3. Results and Discussion

### 3.1. Physicochemical Investigation of the Mucilage

#### 3.1.1. Micromeritic Properties

Micromeritic evaluations on bulk density, tapped density, true density, Carr's index, Hausner's ratio, and angle of repose were done to the powder mucilage to determine the flow properties of the mucilage (Table 1). The bulk and tapped densities of the mucilage powder indicated that there was noticeable reduction in the volumes of the mucilage powder when subjected to tapping pressure. This might be due to the high compaction properties of the powders which are desirable properties in tablet compression [33]. The density of the mucilage is also comparable with the density of water. This would be very useful to use the mucilage as a suspending agent because density difference between water and mucilage is too small to decrease the rate of sedimentation.

Generally, Carr's index values from 5 to 15 and Hausner′s ratio ≤ 1.25 account for powders having good flow properties [34]. Angles of repose ≤ 30° are usually indicative of free flowing materials, while an angle of repose ≥ 40° exhibits poorly flowing material [35]. In this study, Carr's index (%), Hausner's ratio, and the angle of repose of an AEM powder were found to be lower than the values reported in extended release indomethacin capsules and fall within acceptable ranges [36]. This is consistent with the study conducted on micromeritic properties of *Mimosa pudica* seed mucilage as pharmaceutical additives which exhibited good flow characteristics [37]. Thus, it can be inferred that AEM has a good flow property and could have a potential application as an excipient in the preparation of different pharmaceutical dosage forms.

#### 3.1.2. Swelling Power

**Figure 1:**
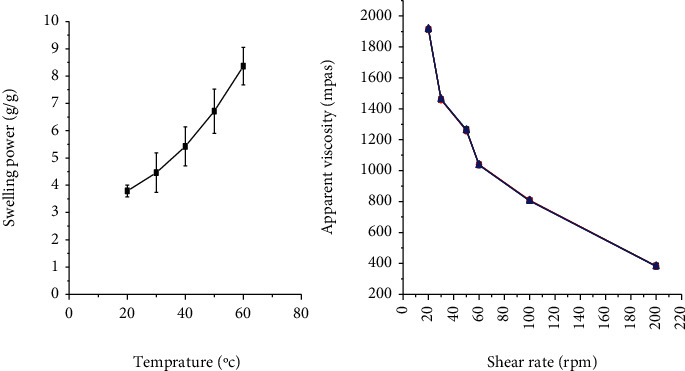
Swelling power and apparent viscosity (mPas) of *Aloe elegans* mucilage.

The swelling power of the mucilage at different temperatures is shown in Figure 1. Its swelling power ranged from 3.79% (20°C) to 8.36% (60°C). The swelling power of the mucilage was significantly increased as a function of temperature [18]. This is in agreement with the study performed on cactus mucilage [38]. The increment in swelling power of the mucilage with temperature could be due to the distortion of the intermolecular bonds of the mucilage molecule with temperature which exposes the functional groups of the mucilage molecule for binding with water molecules.

#### 3.1.3. pH

The pH of the mucilage dispersions at different concentrations was in the range of 4.76 to 5.11. The pH values of the dispersions are comparable with that of *Aloe vera* mucilage having a pH value of 5.24, and they can be categorized under the low acid group (pH > 4.5) [5]. This characteristic of the mucilage is essential for its potential use as a pharmaceutical excipient.

#### 3.1.4. Effect of Shear Rate on Apparent Viscosity

As shown in Figure 1, the apparent viscosity of AEM was decreased significantly (*p* < 0.05) with increasing shear rate. This signifies the pseudoplastic flow nature of the mucilage, which is one of the ideal characteristics of suspending agents that promotes the redispersibility of pharmaceutical suspensions [39]. Similar results were reported on the suspending properties of *Grewia* polysaccharide gum in ibuprofen suspension [40].

#### 3.1.5. Phytochemical Screening of Mucilage

Phytochemical tests were carried out on the *Aloe elegans* mucilage, and the results confirmed the presence of carbohydrates and the absence of other secondary metabolites in the mucilage. The absence of these substances implies that AEM was not contaminated (Table 2). However, the presence of carbohydrates indicates its potential to be utilized as a pharmaceutical excipient [37].

### 3.2. Evaluation of Suspensions

#### 3.2.1. Sedimentation Volume of the Suspensions

**Figure 2:**
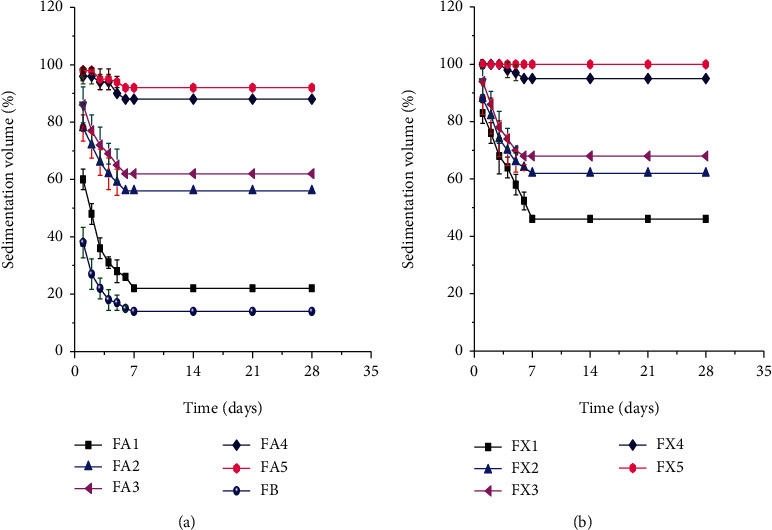
Sedimentation volumes of suspensions containing *Aloe elegans* mucilages (a) and xanthan gum (b) at different concentrations.

The sedimentation volumes (%) of a paracetamol suspension prepared with different concentrations of AEM and XG over a storage period of one month are shown in Figures 2(a) and 2(b). At the 7th day of storage, the sedimentation volumes of FB, FA1, FA2, FA3, FA4, and FA5 were found to be 14%, 24%, 56%, 64%, 88%, and 94%, respectively. Likewise, the sedimentation volumes of FX1, FX2, FX3, FX4, and FX5 were found to be 46%, 64%, 68%, 95%, and 100%, respectively. The sedimentation volumes of all formulations were analyzed using ANOVA. As a result, the sedimentation volumes of the formulations were found to be varied with concentration, time, and types of the suspending agents. Thus, the sedimentation volumes of all formulations prepared with XG and AEM were found to be increased significantly (*p* < 0.05) with an increase in their concentration. Moreover, the results also showed that the sedimentation volume of all suspensions prepared with XG was significantly higher than (*p* < 0.05) those prepared with AEM at the same concentration. This could be due to the variation in viscosity among the suspending agents [27]. However, the sedimentation volumes of all formulations prepared with AEM were significantly higher than (*p* < 0.05) preparations prepared without any suspending agents. This signifies that, the mucilage has a potential to be used as a suspending agent in pharmaceutical suspensions.

The sedimentation volumes of the preparations were found to be decreased significantly (*p* < 0.05) with the time of storage in the first six days in all formulations except FX5 (100%), and then the sedimentation volumes remained constant for about three weeks of storage.

This finding is similar to the results reported in the mucilages of two local *Opuntia* species that were evaluated for their suspending property in comparison with NaCMC in a paracetamol suspension [41]. Thus, AEM has a potential to be utilized as a suspending agent in order to prepare a stable suspension similar to XG-containing formulations.

**Figure 3:**
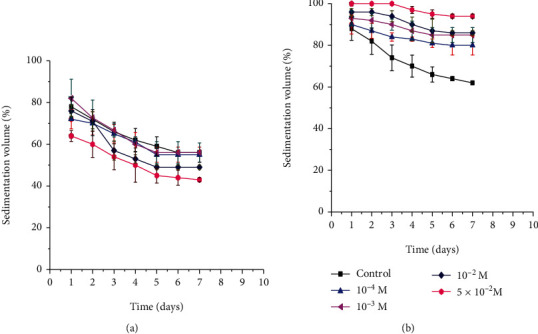
Effect of NaCl concentrations on the sedimentation volumes of suspensions prepared using 3% *Aloe elegans* mucilage (a) and 3% xanthan gum (b) as suspending agents.

*(1) Effect of Electrolyte Concentration on Sedimentation Volume*. The importance of different electrolyte concentrations on the sedimentation volumes of paracetamol suspensions containing AEM and XG at 3% (*w*/*v*) concentration is depicted in Figures 3(a) and 3(b), respectively. In this study, the sedimentation volumes of suspensions that had XG as a suspending agent have shown a significant increase (*p* < 0.05) with the concentration of electrolytes (NaCl). There was a low sedimentation volume in the formulations devoid of electrolytes and followed by formulations that had low NaCl concentration. On the other hand, in the case of a high electrolyte concentration, there was a high sedimentation volume. This might be due to the alteration in zeta potential of the dispersed particles [42]. However, with regard to AEM-based suspensions, a low sedimentation volume of drug particles was obtained in formulations containing a high concentration of electrolytes. This could result from the reduction in the number of bonds between suspended particles [29].

**Figure 4:**
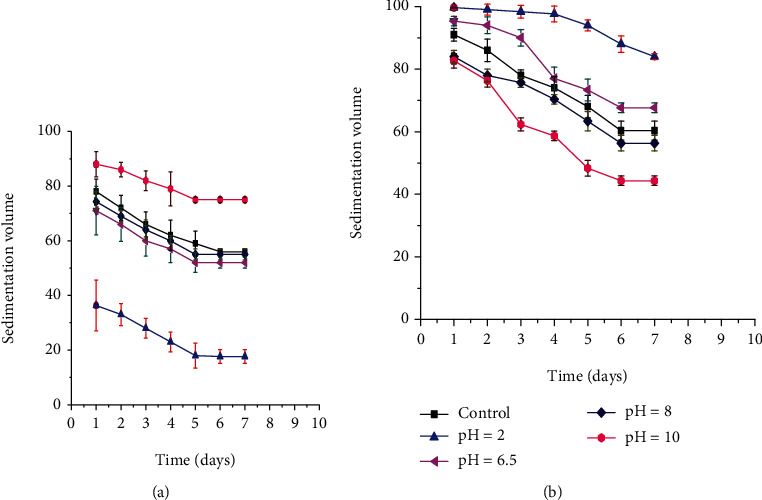
Effect of different pH values on sedimentation volumes of suspensions prepared by 3% *Aloe elegans* mucilage (a) and 3% Xanthan gum (b) as a suspending agent.

*(2) Effect of pH on Sedimentation Volume*. The effect of different pH values (2, 6.5, 8, and 10) on sedimentation volumes of formulations containing 3% AEM and 3% XG is depicted in Figures 4(a) and 4(b), respectively. The sedimentation volumes of formulations containing AEM at the 7th day were 17.67% (pH 2), 54% (pH 6.5), 55% (pH 8), 75% (pH 10), and 56% (control). ANOVA revealed that the differences in sedimentation volumes were statistically significant (*p* < 0.05) among the different pH values and also with that of the control. This lower sedimentation volume at lower pH values might be due to the higher zeta potential of the formulation [37]. Whereas, the sedimentation volume of formulations containing XG at the 7th day were found to be 90% (pH 2), 63% (pH 6.5), 58% (pH 8), 44% (pH 10), and 64% (control). ANOVA for sedimentation volumes indicated that the differences were statistically significant (*p* < 0.05) among the different pH values and also with that of the control (64%) except for the formulation at pH 6.5 (63%). This is in line with the study reported in the literature [26].

#### 3.2.2. Apparent Viscosity of the Suspensions

Apparent viscosity ensures the stability and accuracy of dosing of pharmaceutical suspensions by preventing fast sedimentation of particles and offering the suspension to redisperse easily upon shaking. As the apparent viscosity of the suspension increases, the dispersed phase settles at a slower rate and remains dispersed for a longer period of time [43]. The apparent viscosities of all formulations prepared with AEM and XG were increased significantly (*p* < 0.05) with increments of suspending agent concentration [44, 45]. However, the apparent viscosities of formulations with XG were found to be significantly (*p* < 0.05) higher than that of AEM-containing formulations.

**Figure 5:**
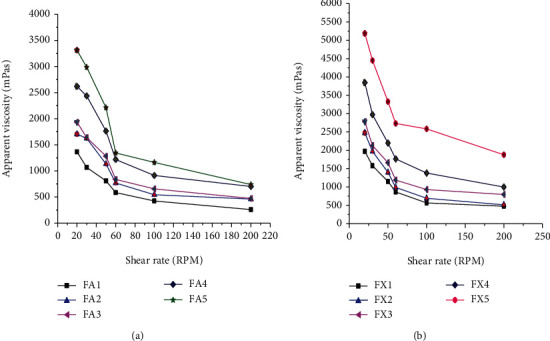
Apparent viscosities of suspensions prepared from *Aloe elegans* mucilage (a) and suspensions prepared from Xanthan gum (b) at different shear rates.

Results of the apparent viscosities plotted against shear rate for all the formulated suspensions are presented in Figures 5(a) and 5(b). As indicated in the figure, the apparent viscosity of the suspensions was decreased significantly (*p* < 0.05) with shear rate in all formulations. This is due to the shear thinning characteristics of the suspensions [37]. This suggests that the suspension will be easily redispersed, and a stable dose can be withdrawn with minimum agitation. This result is similar to the study reported by many experts [45, 46]. Thus, all suspensions formulated with AEM and XG in this study exhibited pseudoplastic flow behavior which is the desirable property of a good suspending agent. Thus, based on the apparent viscosity property, AEM may have a potential to be utilized as suspending agent.

#### 3.2.3. Flow Rate of the Suspensions

The flowability of a suspension is an important quality measurement as it determines ease of withdrawal of the drug from the container. In this study, the flow rates of paracetamol suspensions prepared from different concentrations of AEM as a suspending agent were compared with those of the suspensions prepared using XG. The flow rates of paracetamol suspensions prepared from AEM and XG were found to be decreased significantly (*p* < 0.05) with an increase in the concentration of these suspending agents (Table 3). This is in agreement with the study on *Ocimum basilicum* mucilage [40]. Moreover, the flow rates of AEM-based suspensions were significantly higher (*p* < 0.05) than XG-containing suspensions at all concentrations. The difference in flow rates of the formulations might be due to the variation in viscosity of the suspending agents with their respective concentrations. Hence, AEM can be used as the best suspending agent for suspension formulations.

#### 3.2.4. Redispersibility Rate of Suspensions

Any pharmaceutical suspension produces sediment on storage, and the rate of redispersibility is one of the major quality features of all pharmaceutical suspensions. The number of inversions required to redisperse the suspension formulations after a week and a month is presented in Table 4. The redispersibility rate of suspensions prepared from AEM and XG was decreased significantly (*p* < 0.05) with an increase in the concentration of the suspending agents. This is because the increment in viscosity and the sedimentation volume of the suspension has retarded the terminal velocity of the particles and reduced interparticle attraction thereby making redispersion possible [27]. Moreover, the redispersibility rate of suspensions prepared from AEM was significantly higher (*p* < 0.05) than the one prepared from XG. Thus, suspensions containing XG as a suspending agent were easily redispersed than AEM containing preparations at the same concentration level and time. This could be due to the higher viscosity of the formulations with XG.

However, the redispersibility rates of all formulations were found to be increased significantly (*p* < 0.05) with an increase in storage period. The number of inversions required to redisperse the suspension formulations after a week and a month is presented in Table 4. The number of inversions required to redisperse paracetamol suspensions prepared with XG in concentrations of 1% and 2% was found significantly lower (*p* < 0.05) than those prepared with AEM. However, there was no significance difference (*p* < 0.05) in the rate of redispersibility at concentrations of 3%, 4%, and 5% in both XG- and AEM-containing suspensions. Hence, using concentrations of 3, 4, and 5 *w*/*v* (%) of AEM could be considered as the potential concentrations at which the mucilage would give a good suspending ability.

#### 3.2.5. pH of the Suspensions

The pH values of the paracetamol suspensions were determined at intervals of one week up to 21 days of storage at room temperature. As shown in Tables 5 and 6, the pH values of the suspensions prepared with AEM and XG were found in pH ranges from 5.25-6.23 to 5.15-6.33, respectively. This finding is within the 2013 specifications stated in BP (pH = 5 − 6.5) [40]. However, a significant (*p* < 0.05) variation in pH upon storage was observed among suspensions containing AEM unlike suspensions containing XG. This could be due to the microbial decomposition of the mucilage upon storage [6].

#### 3.2.6. Particle Size

The particle size of the mucilage is assessed because it has significant effects on dissolution, bioavailability, and content uniformity and stability of the drug product. Results of this study indicated that particle size of the mucilage powder was generally increased as the suspending agent concentration is increased as shown in Tables 5 and 6. The proportion of much less fine particles within the mucilage implies that the mucilage may have good flow properties [39].

#### 3.2.7. Assay of the Suspensions

The drug content of all paracetamol suspensions prepared with AEM and XG fell within the acceptable range of the 2013 specifications of BP (Tables 5 and 6) [31].

#### 3.2.8. Dissolution Profile of the Suspensions

Even though the dissolution test is a routine test for quality control of pharmaceutical solid dosage forms, it is also used for suspensions as there may be problems in dissolution of the active ingredient which is the rate limiting step in an absorption process.

**Figure 6:**
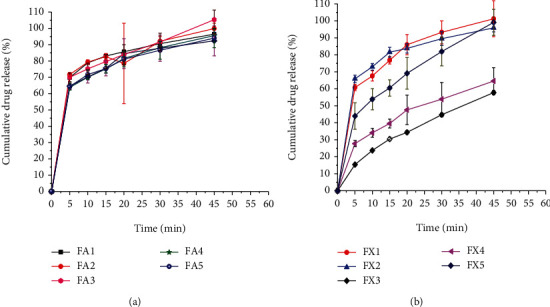
Drug-release profiles of suspension formulations containing different concentrations of *Aloe elegans* mucilage (a) and (b) xanthan gum as suspending agents.

The dissolution profiles of paracetamol suspensions formulated with different concentrations of suspending agents are presented in Figures 6(a) and 6(b). The results revealed that the suspension prepared from AEM released more than 85% of the active ingredient at all concentration levels tested within 30 minutes, while the suspension prepared from XG released more than 85% of the active substance containing 1%, 2%, and 3% of XG only within 45 minutes. For the suspension prepared at concentrations of 4% and 5% XG, the drug-release patterns of the suspension were below the BP acceptance range. The slow release from these formulations could be attributed to the relatively high viscosity of the suspensions as compared to those of the mucilage and might be due to the low agitation speed that provided a lower hydrodynamic force, which generated a slow dissolution rate [43]. The drug-release rate in both AEM- and XG-containing suspensions, however, increased significantly (*p* < 0.05) as the concentration of the suspending agents decreased. This could be partly attributed to the reduction in viscosity as the concentration of the suspending agents decreased. Similar results were reported in the literature [47, 48].

## 4. Conclusion

From this finding, the sedimentation volume, apparent viscosity, flow rate, rate of redispersibility, pH, drug content, and dissolution profiles of formulations prepared with the *Aloe elegans* mucilage showed a potential to be utilized as an alternative suspending agent in pharmaceutical suspensions.

## Figures and Tables

**Table 1:** Powder properties of *Aloe elegans* mucilage.

Properties of *Aloe elegans* mucilage	Results
Bulk density (g/cc)	0.73 ± 0.03
Tapped density (g/cc)	0.79 ± 0.02
Carr's index	8.62 ± 6.23
Hausner's ratio	1.1 ± 0.08
Angle of repose	22.61 ± 1.35

Values are presented as M ± SD; *n* = 3; mean value is significant (*P* < 0.05).

**Table 2:** Phytochemical screening of *Aloe elegans* mucilage.

Identification test	Name of the test	Observation
Carbohydrates	Molisch's test	(+)
Alkaloids	Mayer's reagent and Wagner's test	(-)
Free anthraquinones	10% ammonia solution	(-)
Anthraquinone-O-glycoside	10% ammonia solution	(-)
Anthraquinone-C-glycosides	Ferric chloride test	(-)
Polyphenols	Ferric chloride test	(-)
Flavonoids	Shinoda's reduction test	(-)
Coumarins	10% ammonia solution	(-)
Saponins	Distilled water	(-)
Tannins	1% gelatine	(-)
Steroids	Concentrated sulphuric acid	(-)

(+) means presence and (-) means absence of secondary metabolites.

**Table 3:** Flow rate of suspensions prepared by different concentrations of *Aloe elegans* mucilage and Xanthan gum.

Concentration (% *w*/*v*)	Flow rate of the suspension (ml/sec) (mean ± SD)	
	AEM-containing formulation	XG-containing formulation
1	1.2 ± 0.10^a^	0.43 ± 0.01^b,c^
2	1.08 ± 0.26^a^	0.17 ± 0.02^c^
3	0.71 ± 0.17^b^	0.07 ± 0.03^c^
4	1%5 ± 0.09^b^	Intermediate flow
5	Intermediate flow	No flow

The letters a, b, c, and d indicate significant difference (*p* < 0.05) in flow rates of AEM- and XG-containing formulations at different concentrations. Intermediate flow is when the suspension does not fully flow out of the pipette. No flow is when the suspension does not flow out of the pipette with gravitational force.

**Table 4:** Redispersibility rate of suspensions prepared by *Aloe elegans* mucilage and xanthan gum.

Concentrations	Rate of redispersibility (cycles)			
	After seven days		After one month	
	AEM	XG	AEM	XG
1	16.70 ± 1.53^a^	11.00 ± 1.00^b^	22.33 ± 1.53^d^	14.00 ± 1.00^e^
2	10.00 ± 2.00^b^	7.00 ± 1.00^b^	15.00 ± 2.00^e^	12.00 ± 0.00^e^
3	3.67 ± 0.53^c^	2.67 ± 0.51^c^	6.31 ± 1.53^f^	5.00 ± 0.00^f^
4	2.30 ± 0.58^c^	1.30 ± 0.52^c^	2.30 ± 0.50^c^	4.00 ± 1.00^f^
5	No	No	No	No

The letters a, b, c, d, e, and f show significant difference (*p* < 0.05) among formulations containing different concentrations of suspending agents; No: there is no need to redisperse the suspension (no sediment has formed at the bottom of the container).

**Table 5:** pH, particle size, and assay of suspensions using different concentrations *Aloe elegans* mucilage.

Formulation	pH				Particle size (*μ*g)	Assay (%)
	1st day	7th day	14th day	21st day		
FA1	5.29 ± 0.14^a,b^	5.28 ± 0.15^a,b^	5.25 ± 0.15^c^	5.25 ± 0.15^c^	3.25 ± 0.1^a^	103.39 ± 2.86^a^
FA2	5.58 ± 0.36^a^	5.29 ± 0.29^a,b^	5.28 ± 0.28^b^	5.27 ± 0.29^b^	3.70 ± 0.30^a^	103.86 ± 3.67^a,b^
FA3	5.61 ± 0.42^b^	5.58 ± 0.41^b,c^	5.55 ± 0.40^c^	5.54 ± 0.43^c^	5.40 ± 0.41^b^	104.45 ± 1.22^b,c^
FA4	6.23 ± 0.72^b^	5.9 ± 0.39^b,c^	5.89 ± 0.39^c^	5.89 ± 0.39^c^	6.9 ± 0.35^b^	104.85 ± 1.7^c^
FA5	5.45 ± 0.26^b,c^	5.42 ± 0.28^b,c^	5.41 ± 0.27^d^	5.4 ± 0.28^d^	8.2 ± 0.27^b,c^	98.46 ± 1.32^a^

The letters a, b, c, d indicate significant difference (*p* < 0.05) in pH, particle size, and drug content of *Aloe Elegans* mucilage containing formulations.

**Table 6:** pH, particle size, and assay of suspensions using different concentrations of xanthan gum.

Formulation	pH				Particle size (*μ*g)	Assay (%)
	1st day	7th day	14th day	21st day		
FX1	6.08 ± 0.13^a^	5.88 ± 0.11^a^	5.80 ± 0.11^a^	5.70 ± 0.11^a^	2.33 ± 0.51^a^	97.65 ± 0.69^a^
FX2	6.33 ± 0.14^a^	6.26 ± 0.14^a^	5.84 ± 0.14^a^	5.74 ± 0.14^a^	3.28 ± 0.43^b^	99.84 ± 2.55^a^
FX3	5.71 ± 0.40^a,b^	5.61 ± 0.40^a^	5.47 ± 0.48^a^	5.41 ± 0.48^a^	5.67 ± 0.66^b,c^	104.12 ± 2.63^a^
FX4	5.68 ± 0.37^b^	5.57 ± 0.37^b^	5.51 ± 0.37^b^	5.45 ± 0.37^b^	6.72 ± 0.50^c,d^	104.85 ± 0.96^a,b^
FX5	5.43 ± 0.25^a^	5.24 ± 0.08^a^	5.19 ± 0.08^a^	5.15 ± 0.08^a^	8.94 ± 0.78^d^	97.32 ± 1.10^b,c^

The letters a, b, c, and d indicate significant difference in pH, particle size, and drug content in XG-containing formulations at *p* < 0.05.

## Data Availability

The data used to support the findings of this study are included within the article. Further data supporting the findings of this study are available from the corresponding author upon request.

## Abbreviations

AEM: *Aloe elegans* mucilage
ANOVA: Analysis of variance
BP: British Pharmacopeia
FA: Formulations containing *Aloe elegans* mucilage
FX: Formulations containing xanthan gum
XG: Xanthan gum.
